# Etanercept in the treatment of recalcitrant enteropathic arthritis: a case report

**DOI:** 10.1186/1752-1947-6-10

**Published:** 2012-01-11

**Authors:** Mohd Shahrir Mohamed Said, Sazliyana Shaharir, Sakthiswary Rajalingham, Sheikh Anwar Abdullah, Aizan bin Hassanudin, Ngiu Chai Soon, Mohd Shahdan Shahid

**Affiliations:** 1 Medical Department, UKM Medical Center, Jalan Yaacob Latif, Cheras 56000, Kuala Lumpur, Malaysia; 2 Kg Baru Medical Centre, Kuala Lumpur, Malaysia

## Abstract

**Introduction:**

Enteropathic arthritis is one of the recognized extraintestinal manifestations of inflammatory bowel disease and affects up to 25% of patients. The treatment options for refractory disease were rather limited and ineffective until the arrival of biologic therapy in the last few years. The use of etanercept was unique for this disease.

**Case presentation:**

In this case report, a 58-year-old Malay woman with a 17-year history of ulcerative colitis had persistent left knee effusion and synovitis for seven years, despite remission of the primary disease. She had had multiple courses of systemic and intra-articular steroid that caused significant systemic side effects such as impaired fasting glucose, hypertension, cataract, and weight gain. She also had a total left knee replacement for secondary osteoarthritis. But the left knee synovitis and effusion recurred a month after the total knee replacement, and she was subjected to a total synovectomy the following year. In view of failure of remission despite multiple immunosuppressants (100 mg of azathioprine daily, 1 g of sulfasalazine twice a day, 10 mg of prednisolone daily, and 10 mg of methotrexate weekly), 25 mg of subcutaneous etanercept twice weekly was started. After 5 weeks of treatment, complete resolution of left knee effusion and normalization of the inflammatory markers were shown. This continued up to 12 months of follow-up while our patient was on etanercept and 10 mg of methotrexate weekly. No relapse or serious side effects were noted.

**Conclusions:**

This case demonstrates the efficacy of etanercept in recalcitrant enteropathic arthritis with no relapse of the underlying colitis while on treatment. The usage of this tumor necrosis factor inhibitor was unique in this case of rheumatology and gastroenterology.

## Introduction

Enteropathic arthritis is the most common extraintestinal manifestation of inflammatory bowel disease (IBD) and can affect up to 30% of patients [1]. It is a subset of spondyloarthritides and seronegative arthritis, which is a group of inflammatory joint disease that usually affects the enthesis or site of attachment of ligaments and tendons to the bones [2]. There were three principal forms of arthritis associated with IBD: the peripheral, the axial, and a form overlapping between the two [3]. For peripheral arthritis, two types have been described in the literature: oligoarticular (type I) and polyarticular (type II) [4]. Type I usually involves large joints of lower limbs and is associated with IBD flares, whereas type II arthritis is polyarticular and symmetric, usually evolves in chronic disease, and involves not only hands and feet but also large joints [4]. Type II may precede IBD symptoms, and its course is independent of IBD flares.

Corticosteroids are effective in acute cases; however, in some refractory cases, the successful use of disease-modifying anti-rheumatoid drugs (DMARDs) is rather limited. Although there are no randomized controlled trials on the efficacy of sulfasalazine, it is the most widely used in enteropathic spondyloarthritis [5]. Methotrexate and azathioprine are not as well studied and often are ineffective [6]. Also, there were no reports of regression of the enthesitis pathology in spondyloarthropathies with the above DMARD therapies [7]. Analgesics, particularly non-steroidal anti-inflammatory drugs (NSAIDs) and cyclo-oxygenase-2 inhibitors, should be used cautiously as they can exacerbate bowel symptoms [5]. The recent introduction of anti-tumor necrosis factor-alpha (anti-TNF-α) has emerged as a promising therapeutic opportunity and should be considered early in patients whose condition is not sufficiently controlled with DMARDs or in those with axial diseases.

In this case report, a very recalcitrant IBD-related arthropathy or enteropathic arthritis is described in a patient with ulcerative colitis that finally responded to etanercept. The use of this type of TNF-α was unique for this disease. The literature reviews in regard to the use of biologics in enteropathic arthritis will be discussed further in this case report.

## Case presentation

A 58-year-old Malay woman had ulcerative colitis that was diagnosed 21 years ago when she presented with recurrent diarrhea. Five years ago, she started to develop peripheral polyarthritis. There were no other extra-articular manifestations such as uveitis or pyoderma gangrenosum. She had multiple relapses of colitis with polyarthritis and therefore had received multiple courses of tapering-dose oral prednisolone with maintenance of 1 g of mesalazine three times a day and subsequently 100 mg of azathioprine once daily. Finally, two years later, with the above treatment, the primary disease managed to be brought to remission.

In spite of the clinical and histological remission of the IBD, the left knee arthritis seems to be persistent, but other peripheral joints mentioned above were in remission without any permanent deformity. A clinical assessment revealed that the left knee was swollen, warm, and tender. The inflammatory marker C-reactive protein was increased to 1.14 mg/dL (normal is less than 0.5 mg/dL). A knee aspirate revealed clear synovial fluid and did not yield any growth. She received two courses of 500 mg of intravenous methylprednisolone daily for three days followed by a tapering dose of oral prednisolone for one year for active and persistent left knee arthritis, but the effects were temporary. Mesalazine was changed to 1 g of sulfasalazine twice a day, and the azathioprine dose was subsequently increased to 150 mg once daily. Unfortunately, she developed leukopenia; therefore, the azathioprine dose had to be reduced back to 100 mg daily. Despite aggressive treatment with multiple courses of high-dose systemic and local steroid with the maximum tolerated doses of sulfasalazine and azathioprine, she still had recurrent left knee effusion and synovitis. Indeed, the disease had caused significant pain and restriction of mobility. Also, after almost two years of corticosteroid therapy, our patient has endured significant iatrogenic morbidity, including Cushingoid habitus, impaired fasting glucose, hypertension, and bilateral cataract with increased intraocular pressure.

**Figure 1:**
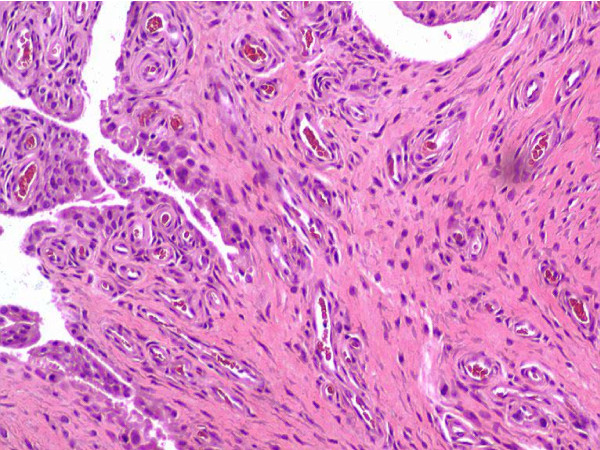
**Synovial tissue of the left knee (hematoxylin and eosin staining, ×400)**. A fragment of fibrocollagenous connective tissue stroma admixed with fibrinous exudates and abundant foci of vascular proliferation indicates an inflammatory process.

In view of persistent unbearable pain and swelling of the left knee with severe secondary osteoarthritis, a total left knee replacement was done in the same year. During the operation, there was active synovitis of the left knee, and a histopathological examination of the synovial tissue revealed 'rice bodies', which are fibrocollagenous connective tissue stroma admixed with fibrinous exudates and abundant foci of vascular proliferation, suggestive of inflammatory arthritis (Figure 1). One month after the operation, the left knee effusion recurred and there was persistent elevation of the inflammatory markers. Subsequently, a left knee arthroscopic debridement and near-total synovectomy were performed five years ago. Ten milligrams of oral methotrexate weekly was added one month after the operation, on top of 1 g of sulfasalazine twice a day, 10 mg of prednisolone once daily, and 100 mg of azathioprine once daily. Unfortunately, despite the above treatment for four months, our patient continued to show poor response and persistent recurrence of knee effusion. Subsequently, 25 mg of subcutaneous etanercept twice weekly, together with the other DMARDs and azathioprine, was initiated after three months.

Eventually, the joint showed resolution of knee joint swelling and synovitis at the fifth week of treatment, visual analogue scale score improved from 70 to 20 mm, and the C-reactive protein level decreased to 0.8 mg/dL. After six months of treatment with etanercept, azathioprine was stopped and sulfasalazine was changed to 1 g of mesalazine three times a day as our patient had a mild flare of ulcerative colitis with community-acquired pneumonia. The steroid was also tapered off after six months of treatment.

Twenty-five milligrams of subcutaneous etanercept biweekly and 10 mg of methotrexate weekly were continued, and after 12 months of follow-up, the colitis or arthritis was in remission and no serious side effects were noted.

## Discussion

The above case demonstrates the challenges in managing enteropathic arthritis given that the therapeutic options for patients with the more severe form have been rather limited. Commonly, oligoarthritis associated with IBD is associated with disease activity; however, in 10% of cases, it evolves into chronic arthritis [4] as in this case. Even though sulfasalazine seems to be promising in IBD-related arthropathy [8], our patient did not show a good response with the treatment, indicating the need for biologic therapy.

Although biologic treatment is a promising treatment option, the trials of this agent involved mainly other spondyloarthropathies, particularly ankylosing spondylitis (AS) and psoriatic arthritis [9-11]. No randomized controlled trials have investigated therapeutic options in IBD-related arthropathy. The treatment is based almost entirely on extrapolation from treatment of other forms of spondyloarthropathies since they shared the same pathogenesis, which involves significant expression of TNF-α in the joint spaces as the major inflammatory mediators.

Among the biologic agents, infliximab, which is a chimeric anti-TNF-α monoclonal IgGI antibody, is the most widely used and has been proven in many studies to control the primary IBD and associated arthritis [12,13]. For etanercept, only one small study by Marzo-Ortega and colleagues [14] showed its efficacy in various spondyloarthritides, including IBD. In that study, the authors reported a significant increase in mean bone mineral density at the hip and spine in all patients who were treated with a six-month course of 25 mg of subcutaneous etanercept biweekly [14]. Etanercept may improve joint symptoms, but studies do not show any benefit for the bowel inflammation as the treatment progresses [15]. It has been proposed that the differential effects of infliximab and etanercept in IBD are due to the inability of the etanercept to bind directly to the cell surface of macrophages causing different signal was transmitted into the cell via transmembrane TNF. These signals do not induce mononuclear cells apoptosis within the bowel mucosa, which is the key pathogenesis in IBD [15]. Although adalimumab has also been shown to be effective for the treatment of AS [16], no trials have specifically examined the efficacy of adalimumab for patients with concomitant IBD and arthritis. Table 1[17-21] summarizes the previous studies of anti-TNF in enteropathic arthritis.

**Table 1:** Case series and studies of biologics in enteropathic arthritis

Study	Number of patients	Study protocol	Outcome
Ellman et al. [17] Open-label	four (treatment refractory peripheral arthritis)	Infliximab 5 mg/kg	100% improvement of arthritis

Van den Bosch et al. [13]	four CD	Infliximab	100% improvement in axial and peripheral arthritis

Herfarth et al. [18] Open-label	59 (peripheral arthritis refractory to corticosteroids, 6-mercaptopurine, azathioprine, or methotrexate)	Infliximab 5 mg/kg at 0 (luminal CD) or 0, two, and six weeks (fistulizing CD)	61% (35/59) improvement of 1 point in the arthritis component of the Crohn disease activity index (CDAI) score at 12 weeks and complete resolution of arthritis in 27/59 patients (46%)

Kaufman et al. [19] Open-label	22 CD one ulcerative colitis 11 inflammatory arthralgias (three active synovitis) 11 axial/sacroilitis	Infliximab 5 mg/kg	After two weeks, partial improvement in duration of morning stiffness, tender joint count, and visual analogue scale for pain in 7/11 patients. Clear improvement in frank arthritis in one out of three patients

Van den Bosch et al. [20] Randomized, double-blind, placebo-controlled trial	40 (SpA with or without inflammatory bowel disease) versus placebo	Infliximab 5 mg/kg at 0, two, and six weeks or placebo	At 12 weeks, improvement in patient and physician global assessment (18 in infliximab group versus 69 in placebo group, *P *< 0.001) and physician global assessment (16.5 versus 72, *P *< 0.001).

Generini et al. [21] Randomized controlled study	24 CD with SpA (16 active CD and eight inactive CD) versus 12 control subjects (active CD treated with corticosteroids, azathioprine, salicylates, and antibiotics)	Infliximab 5 mg/kg at 0, two, and six weeks followed by 3 mg/kg (inactive CD) or 5 mg/kg (active CD) every five to eight weeks	Improvement of enthesitis or BASDAI or both at six, 12, and 18 months in all patients

Marzo-Ortega et al. [14]	10 (7 ankylosing spondylitis, two CD, and one undifferentiated SpA)	Subcutaneous etanercept 25 mg biweekly for 24 weeks	All patients achieving greater than 50% improvement of BASDAI at four weeks and throughout study period

BASDAI, British ankylosing spondylitis disease activity index; CD: Crohn disease; SpA: sponyloarthropathy.

Although etanercept is not effective for bowel symptoms in IBD, it was started in this patient because she preferred to administer the subcutaneous medications herself at home and because her underlying colitis was in remission. Although there has been a concern of a potential flare of underlying IBD with etanercept, [22] our patient had no relapse of colitis after 12 months of treatment and the underlying arthritis remained in remission.

Despite all of the potential benefits of the biologics, we still have an unresolved issue pertaining to the duration of the treatment. In other spondyloarthritis such as AS and psoariatic arthritis, a sustained response of up to two years was reported [14,23]. Unfortunately, most of these patients relapse after a few weeks of withdrawal of the treatment. The relapse rate is high, ranging from 75% to 100%, and the mean time to relapse was reported to be as early as six to 17.5 weeks. However, retreatment is safe and still effective, resulting in similar clinical improvement [24]. Therefore, the optimum duration of the treatment is not known, and most likely our patient will require lifelong and extremely expensive treatment.

In Malaysia, the cost and feasibility of biologics are indeed the major concern, and the treatment is available only in large tertiary centers. To date, only a few studies have looked into the cost-effectiveness of etanercept among patients with other groups of seronegative arthritis such as AS and psoariatic arthritis. The primary outcome measure used was quality-adjusted life years (QALYs), which was derived from utility values estimated as function disability measured by the British ankylosing spondylitis disease activity index (BASDAI), British ankylosing spondylitis functional index (BASFI), and EQ-5D scores. The results were rather conflicting and inconclusive as different populations were involved with different criteria of each patient, the time the TNF blocker was started was not the same, and no randomized control trials directly compared various anti-TNF-α inhibitors. A study by Ara and colleagues [25] demonstrated the potential cost-effectiveness of etanercept plus NSAIDs compared with NSAIDs alone in patients who had severe unremitting AS and who failed two optimum NSAIDs in the UK, and the majority of costs were below £25,000 per QALY at five years. Etanercept is the only biologic within the range of cost-effectiveness estimates considered by the National Institute for Health and Clinical Excellence to represent a good value for money in the National Health Service [25]. On the other hand, the incremental cost-utility ratios in The Netherlands vary between £43,000 and £124,000 per QALY for etanercept compared with usual care and £67,000 to £237,000 for infliximab [26]. Thus, this model suggests that the high drug costs restrict efficient use in all patients who have a BASDAI of more than 4. Bravo and colleagues [27] demonstrated, among patients with psoariatic arthritis, that, at a 10-year time horizon, etanercept was more cost-effective than infliximab. The incremental cost-effectiveness ratio was significantly higher at £26,000 per QALY to £31,000 per QALY for etanercept compared to £165,000 to 205,000 per QALY in infliximab with no superiority effectiveness. However, this study was not a head-to-head comparison.

Even though biologic treatment has demonstrated marked clinical and radiological improvement in both synovitis and enthesitis, thus preventing ankylosis in the short term, it remains to be shown whether patients benefit from long-term anti-TNF therapy and whether radiologic progression and ankylosis can be stopped totally. A study by Baraliakos and colleagues [28] showed that, after two years of treatment, magnetic resonance imaging revealed that minor spinal inflammation was still present in 64% of the patients. Therefore, long-term treatment may not guarantee the prevention of ankylosis, but at least it can delay the process and, hopefully with longitudinal studies of early disease, will be able to answer the question of whether these therapies constitute true long-term disease modifiers in spondyloarthropathies.

## Conclusions

Etanercept seems to be promising in controlling the disease, but the long-term cost-effectiveness and its side effects are yet to be proven. Further studies of these biologic therapies need to be continued and hopefully will offer better options to patients.

### Consent

Written informed consent was obtained from the patient for publication of this case report and any accompanying images. A copy of the written consent is available for review by the Editor-in-Chief of this journal.

## Abbreviations

AS: ankylosing spondylitis; BASDAI, British ankylosing spondylitis disease activity index; DMARD: disease-modifying anti-rheumatoid drug; IBD: inflammatory bowel disease; NSAID: non-steroidal anti-inflammatory drug; QALY: quality-adjusted life year; TNF-α: tumor necrosis factor-alpha.

## Competing interests

The authors declare that they have no competing interests.

## Authors' contributions

SS wrote the case report and interpreted the patient data with MSS. All of the other authors involve in the management of this patient. All authors have read and approved the final manuscript.
